# Pretreatment screening for distant metastases in the Dutch head and neck centers: 10 years later

**DOI:** 10.1007/s00405-016-3897-5

**Published:** 2016-01-14

**Authors:** Asaf Senft, Otto S. Hoekstra, Jonas A. Castelijns, C. René Leemans, Remco de Bree

**Affiliations:** 1 Department of Otolaryngology-Head and Neck Surgery, VU University Medical Center, Amsterdam, The Netherlands; 2 Department of Radiology and Nuclear Medicine, VU University Medical Center, Amsterdam, The Netherlands; 3 Department of Head and Neck Surgical Oncology, UMC Utrecht Cancer Center, Utrecht, The Netherlands

**Keywords:** Distant metastases, Screening, Survey, Indications, Diagnostic techniques

## Abstract

To evaluate the current practice and change in practice concerning screening for distant metastases in head and neck squamous cell carcinoma patients, we performed a survey with the same questionnaire as 10 years ago among the eight centers of the Dutch Head and Neck Society treating head and neck cancer in The Netherlands. Factors related to extensive lymph node metastases are the most frequent indication for screening for distant metastases. The combinations of whole body PET-CT and contrast-enhanced chest CT are nowadays the diagnostic techniques for routinely screening for distant metastases. Screening for distant metastases is performed more frequently than 10 years ago. Although the sensitivity of the diagnostic pathway needs to be improved, most centers are satisfied with the current diagnostic pathway. A reduction of variation in indications and diagnostic techniques used for screening for distant metastases is observed during the last 10 years. In future guidelines patients’ selection and diagnostic tests need to be specified in more detail.

## Introduction

Head and neck squamous cell carcinomas (HNSCC) have a tendency to metastasize to regional lymph nodes rather than to spread hematogeneously to distant sites. The incidence of distant metastases is directly related to the stage of the tumour, particularly the presence and extension of lymph node metastases, and regional control above the clavicles. Once distant metastases have been detected, the prognosis is dismal. The median time to death from the diagnosis of distant metastases ranges 1–12 months. About 88 % of patients with distant metastases will die within 12 months. Thus, the detection of distant metastases is critical for prognostication and for the choice of treatment in patients with HNSCC. Patients with known distant metastatic disease can possibly be spared the toxicities of aggressive and often unnecessary locoregional therapy [1].

Ten years ago, we performed a survey which showed a substantial variation in indications and diagnostic techniques used for pretreatment screening for distant metastases between the major institutions treating head and neck cancer in The Netherlands. Eight of 19 (42 %) clinicians stated that they were not satisfied with the current course of diagnostic investigations, because of a perceived lack of sensitivity of the current tests [2]. In these 10 years, diagnostic techniques improved and PET-CT became wider available.

Since then an update of the Dutch guidelines on laryngeal carcinoma (version 3.0, 2010) of the Dutch Head and Neck Society (NWHHT) was published (oncoline.nl) in which it was stated that screening by chest CT was indicated in patients with three or more lymph node metastases, low jugular metastases and N2c or N3 disease. In the recent version of the Dutch NWHHT guidelines for head and neck cancer it is advised to perform FDG-PET-CT in high risk HNSCC patients.

To evaluate the current practice and change in practice concerning the diagnostic work-up in HNSCC patients, we performed a survey with the same questionnaire as 10 years ago among the eight centers of the Dutch Head and Neck Society treating head and neck cancer in The Netherlands.

## Materials and methods

Ethical considerations: no ethical approval was needed for this survey on the routine clinical practice.

**Fig. 1:**
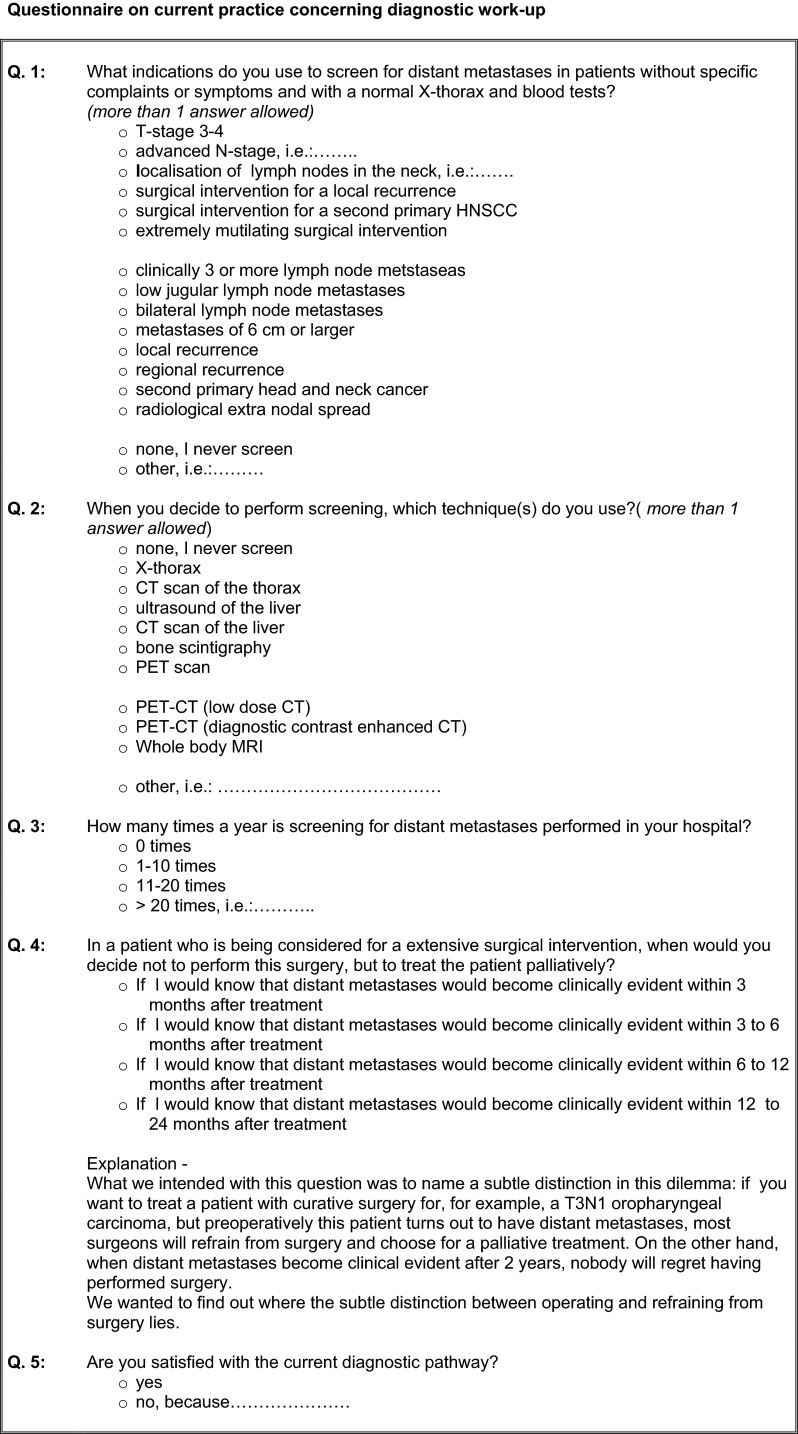
Questionnaire on current practice concerning diagnostic work-up

The questionnaire on current clinical practice concerning screening for distant metastases in HNSCC patients was sent to eight head and neck surgeons as representatives of the eight head and neck centers of the Dutch Head and Neck Society (NWHHT) treating head and neck cancer in The Netherlands. The questionnaire (Fig. 1) was accompanied by an explanatory mail.

## Results

The response rate was 100 %. Indications for screening for distant metastases are summarized in Table 1. In Table 2 indications for screening for distant metastases related to lymph node metastasis were specified. In one center all N+ patients undergo screening for distant metastases. The results of the question which techniques (besides chest X-ray) are routinely used for screening are shown in Table 3.

**Table 1:** Results relating to question about indications for screening for distant metastases

Indication	Responders		Specifications	
	2005 (*n* = 19)	2015 (*n* = 8)	2005	2015
Lymph node metastasis	12/19 (63 %)	8/8 (100 %)	≥N2b, levels, IV–V, supraclavicular	See Table 2
Extremely mutilating surgical intervention	11/19 (58 %)	5/8 (63 %)		
Local and/or regional recurrence	9/19 (47 %)	4/8 (50 %)		
T-stage 3–4	6/19 (32 %)	1/8 (13 %)		
Second primary head and neck cancer	4/19 (21 %)	3/8 (38 %)

**Table 2:** Indications for screening for distant metastases related to lymph node metastasis

Indication	Responders (*n* = 8)
Advanced *N*-stage (N2–N3)	5^a^ (63 %)
Localisation of lymph nodes in the neck (Level V)	4 (50 %)
Clinically three or more lymph node metastases	6 (75 %)
Low jugular lymph node metastases	7 (88 %)
Bilateral lymph node metastases	7 (88 %)
Metastases of 6 cm or larger	8 (100 %)
Regional recurrence	3 (38 %)
Radiological extra nodal spread	2 (25 %)

^a^In one center not N2a

**Table 3:** Results relating to question which techniques are routinely used besides chest X-ray

Diagnostic technique	Responders	
	2005 (*n* = 19)	2015 (*n* = 8)
Contrast enhanced chest CT	16/19 (84 %)	7/8 (88 %)
Ultrasound liver	10/19 (53 %)	
CT liver	3/19 (16 %)	
Bone scintigraphy	8/19 (42 %)	
PET(-low dose CT)	13/19 (68 %)^a^	8/8 (100 %)^b^

^a^Only in research protocol

^b^In one center only in selected cases

Two (25 %) clinicians reported screening in 11–20 patients annually and 6 (75 %) performed screening for distant metastases in more than 20 patients.

If a patient with HNSCC could only be cured by extensive surgery, the number of clinicians that would have refrained from curative surgery and resorted to palliative measures if they considered that the patient would develop distant metastases, within a certain period was 7 (88 %) for distant metastases within 3 months after surgery, 7 (88 %) for 3–6 months, 6 (75 %) for 6–12 months and 2 (25 %) for 12–24 months after surgery. One center could not answer this question because it “depends on many factors like actual complaints caused by the tumor, co-morbidity, patient preferences, expected functional outcome of the procedure, etc.”.

Six (75 %) centers were satisfied with the current diagnostic pathway. Two (25 %) centers stated that they were not satisfied with the current course of diagnostic investigations, because “Dilemma between routinely performing chest X-ray or CT (in head and neck cancer patients in general)” and “Financial problems (like to do more chest CT and/or PET-CT)”.

## Discussion

In 10 years’ time the clinical practice of screening for distant metastases has changed: extensive lymph node metastases is the main indication for pretreatment screening of distant metastases, FDG-PET-CT combined with contrast-enhanced chest CT is the current screening technique and most centers are satisfied with current diagnostic pathway.

The incidence of distant metastases from HNSCC at presentation is generally too low to warrant routinely extensive radiological screening for distant metastases in all HNSCC patients. Therefore, high risk factors have been identified and validated: three or more lymph node metastases, bilateral lymph node metastases, lymph nodes larger than 6 cm, low jugular lymph node metastases, regional tumour recurrence and second primary tumours [3, 4]. Another radiological high risk factor is extra nodal spread [5]. Most of the centers use these criteria, although some centers simplified these factors using N2-N3 disease as indication for screening for distant metastases. Some indications do not harbor a high risk of distant metastases, but may be justified if the morbidity of a planned treatment or burden to the patient is very high, e.g., extremely mutilating surgery.

While 10 years ago several diagnostic techniques were used, currently PET-CT and contrast enhanced chest CT are the only techniques and are used in almost all centers routinely. This combination of PET-CT and contrast-enhanced chest CT is the best strategy to screen for distant metastases [6, 7]. In a meta-analysis Xu et al. [8] found for integrated PET-CT a pooled sensitivity and specificity to detect distant metastases of 88 and 95 %, respectively. However, about half of the high risk patients develop distant metastases during follow-up, despite negative screening by PET-CT. Therefore, room for improvement remains. Due to technical improvement whole body MRI is feasible [9] and studies in these high risk HNSCC patients comparing this new technique with the current best technique, i.e., PET-CT (including contrast enhanced chest CT), are needed.

All centers would refrain from extensive treatment if a HNSCC patient would develop clinically manifest distant metastases within 6 months, except one center which makes the decision to treat with curative intent dependent on many factors like actual complaints caused by the tumor, co-morbidity, patient preferences and expected functional outcome of the procedure. Almost all centers would only offer treatment with curative intent if development of distant metastases are expected not to be within 12 months.

Pretreatment screening for distant metastases is performed more frequently: 75 % of head and neck centers more than 20 times a year, in comparison with 26 % of clinicians 10 years ago. Ten years ago 42 % of the clinicians stated that they were not satisfied with the course of diagnostic investigations, because of a perceived lack of sensitivity of the tests at that moment. Although nowadays the sensitivity of the best diagnostic technique, i.e., PET-CT, is still limited, none of the centers mentioned to be dissatisfied by the performance of the diagnostic tests. One center was not satisfied because of the dilemma to perform routinely chest X-ray or CT. However, plain chest X-ray films detect only a minority of all malignant pulmonary lesions detected by CT. Another center has a financial problems with this diagnostic pathway, because the physicians like to do more chest CT and/or PET-CT. Although FDG-PET is an expensive diagnostic test, the detection of distant metastases can avoid futile expensive treatments. When applied in the pre-treatment work-up of high risk HNSCC the addition of FDG-PET did not lead to additional costs [10]. Moreover, PET-CT is nowadays commonly used for radiation treatment planning.

Through the response rate of 100 % and the centralized care for head and neck cancer patients the clinical practice the entire Netherlands is covered by this survey. The same questionnaire as 10 years ago was used making comparison possible.

In the previous survey individual physicians from all eight centers instead of one representative per center were asked limiting direct comparison between both surveys to some extent.

This survey shows a reduction of variation in indications and diagnostic techniques used for screening for distant metastases between the Dutch centers treating head and neck cancer in The Netherlands over the last 10 years. Although the sensitivity of FDG-PET-CT is limited the physicians in most centers are satisfied with the policy to screen HNSCC patients with extensive lymph node involvement routinely by whole body FDG-PET-CT and contrast-enhanced chest CT. In future guidelines patients’ selection and diagnostic tests need to be specified in more detail.
